# Synthesis, Spectroscopic and Theoretical Studies of New Quaternary *N*,*N*-Dimethyl-3-phthalimidopropylammonium Conjugates of Sterols and Bile Acids

**DOI:** 10.3390/molecules19044212

**Published:** 2014-04-03

**Authors:** Bogumil Brycki, Hanna Koenig, Iwona Kowalczyk, Tomasz Pospieszny

**Affiliations:** Laboratory of Microbiocide Chemistry, Faculty of Chemistry, Adam Mickiewicz University, Grunwaldzka 6, Poznań 60-780, Poland; E-Mails: koenig@amu.edu.pl (H.K.); iwkow@amu.edu.pl (I.K.)

**Keywords:** sterols, bile acids, quaternary ammonium salt, conjugates, *N*,*N*-dimethyl-3-phthalimidopropylamine, PASS, PM5 calculations, B3LYP *ab initio* methods

## Abstract

New quaternary 3-phthalimidopropylammonium conjugates of steroids were obtained by reaction of sterols (ergosterol, cholesterol, cholestanol) and bile acids (lithocholic, deoxycholic, cholic) with bromoacetic acid bromide to give sterol 3β-bromoacetates and bile acid 3α-bromoacetates, respectively. These intermediates were subjected to nuclephilic substitution with *N*,*N*-dimethyl-3-phthalimidopropylamine to give the final quaternary ammonium salts. The structures of products were confirmed by spectral (^1^H-NMR, ^13^C-NMR, and FT-IR) analysis, mass spectrometry (ESI-MS, MALDI) as well as PM5 semiempirical methods and B3LYP *ab initio* methods. Estimation of the pharmacotherapeutic potential has been accomplished for synthesized compounds on the basis of Prediction of Activity Spectra for Substances (PASS).

## 1. Introduction

Steroids are a large class of organic compounds. They play a very important role in animals, plants and microorganisms. The best known steroid is certainly cholesterol. Cholesterol was isolated for the first time from gall stones nearly two centuries ago by Chevreul [1,2]. This sterol is an important component of mammalian cell membranes; it is also present in significant concentrations in the brain and nervous tissue [3,4,5,6]. Cholesterol is the biosynthetic precursor of steroid hormones, bile acids, vitamin D and lipoproteins [7,8,9]. Like the functions of cholesterol in mammals, ergosterol is necessary to support the life of fungi. It serves two main purposes: a bulk membrane function and a sparking function. Ergosterol is a biological precursor to vitamin D_2_ (ergocalciferol) [10,11,12].

All sterols are crystalline compounds with a secondary hydroxyl group in the position C(3) of the steroid skeleton, one or two double bonds and differently modified side chains. Rings A/B of the steroid skeleton may have *trans* geometry (the allo series) or *cis* (the normal series). Sterols have the hydroxy group on the C(3) position forming a number of β-sterols with respect to the average plane of the ring. By contrast bile acids have hydroxy groups on the C(3) position which prefer the α orientation [13,14,15,16,17].

Bile acids are major metabolites of cholesterol, being end products of its metabolism in the liver [18,19]. They are isolated from the bile of higher animals, where they are found as sodium salts of peptide conjugates with glycine and taurine. The most important are the primary bile acids, e.g., chenodeoxycholic acid and cholic acid, which are successively transformed into secondary bile acids such as ursodeoxycholic, deoxycholic and lithocholic acids [20,21,22]. Bile acids (e.g., lithocholic, deoxycholic and cholic) are very interesting because they display a large, rigid, and curved skeleton. Moreover, they possess chemically different polar hydroxy groups (3α, 3α,7α and 3α,7α,12α) and amphiphilic properties. Modifications of the functional groups of sterol molecules allow one to obtain systems with high pharmacological activity [23].

Quaternary alkylammonium salts play an important role in the living organisms and many functions of prokaryotic and eukaryotic cells have been shown to be alkylammonium salts dependent [24,25]. These compounds also exhibit excellent antimicrobial activity, and therefore they are used as antiseptics, bactericides and fungicides, as well as therapeutic agents. In general, quaternary alkylammonium salts with good antimicrobial activities contain one or two alkyl chains with lengths in the C_8_–C_14_ range. For the applications as softeners and hair conditioning agents hydrocarbon chain lengths between C_16_ and C_18_ are used [26,27,28]. Phthalimides, and *N*-substituted phthalimides are also an important class of compounds because of their biological activities as antimicrobial agents [29,30]. It has recently been shown that tetrachlorophthalimide derivatives are good α-glucosidase inhibitors [31]. The use of microbiocides of the same type for a long time may cause an increase of resistance of microorganisms to the chemicals used, which is a very serious and dangerous problem. Antimicrobial resistance of bacteria comprises a wide variety of biochemical mechanisms and processes that allow microorganisms to grow in the presence of microbiocides [32,33,34]. There are many ways to overcome the risk of an increasing resistance of microorganisms, however the best one is a periodically application of new microbiocides with modified structures [35,36]. Therefore, connections of sterols and bile acids with various amines or polyamines appears to be an unusually interesting potential approach to such new structures [37,38,39,40].

## 2. Results and Discussion

In the present work, the synthesis and physicochemical properties of some new quaternary *N*,*N*-dimethyl-3-phthalimidopropylammonium conjugates of sterols (ergosteryl 3β-bromoacetate (**4**), cholesteryl 3β-bromoacetate (**5**), dihydrocholesteryl 3β-bromoacetate (**6**)) and derivatives of bile acids (methyl litocholate 3α-bromoacetate (**13**), methyl deoxycholate 3α-bromoacetate (**14**) and methyl cholate 3α-bromoacetate (**15**)) with *N*,*N*-dimethyl-3-phthalimidopropylamine in acetonitrile are investigated.

**Scheme 1:**
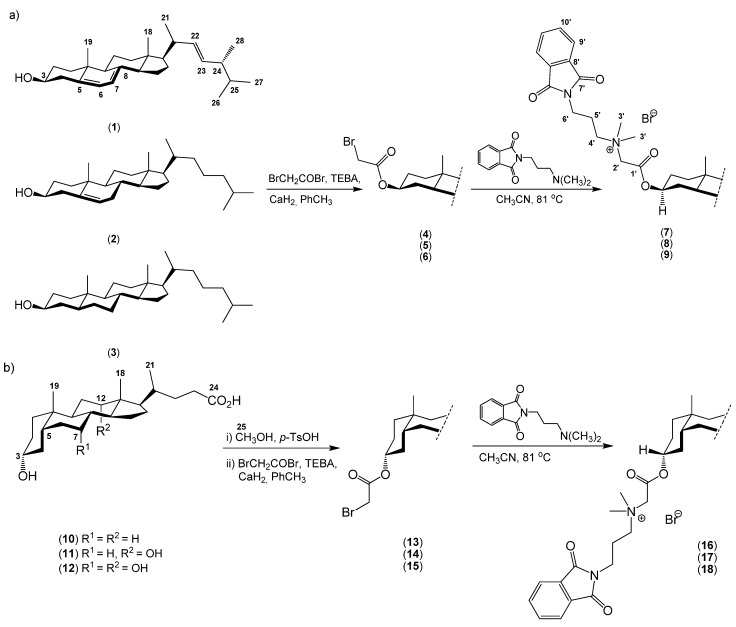
Synthesis of quaternary phthalimidopropylammonium conjugates of sterols **7**–**9** (**a**) and bile acids **16**–**18** (**b**).

New quaternary 3-phthalimidopropylammonium conjugates of steroids were obtained by reaction of ergosterol (**1**), cholesterol (**2**), cholestanol (5α-cholestan-3β-ol, **3**), and bile acids **10**–**12** with bromoacetic acid bromide to give **4**–**6** and **13**–**15**. The 3β-bromoacetates of sterols and 3α-bromoacetates of bile acids, as well as *N*,*N*-dimethyl-3-phthalimidopropylamine were prepared according to the literature procedures [41,42]. The structure of products was confirmed by ^1^H-NMR, ^13^C-NMR, and FT-IR analysis, as well as ESI-MS and MALDI. The syntheses of conjugates **7–9** and **16**–**18** are shown in Scheme 1.

Potential pharmacological activities of the synthesized compounds have been determined on the basis of computer-aided drug discovery approach with *in silico* Prediction of Activity Spectra for Substances (PASSs) program. It is based on a robust analysis of the structure-activity relationship in a heterogeneous training set currently including about 60,000 biologically active compounds from different chemical series with about 4,500 types of biological activities. Since only the structural formula of the chemical compound is necessary to obtain a PASS prediction, this approach can be used at the earliest stages of investigation. There are many examples of the successful use of the PASS approach leading to new pharmacological agents [43,44,45,46,47]. The PASS software is useful for the study of biological activity of secondary metabolites. We have selected the types of activities that were predicted for a potential compound with the highest probability (focal activities). If predicted activity is higher than 0.7 (PA > 0.7), the substance is very likely to exhibit the activity in experiment and the chance of the substance being the analogue of a known pharmaceutical agent is also high. If predicted activity is between 0.5 and 0.7 (0.5 < PA < 0.7), the substance is unlikely to exhibit the activity in experiment and the similarity to known pharmaceutical substance is very limited.

The structures of all synthesized compounds were determined from their ^1^H- and ^13^C-NMR, FT-IR and ESI-MS spectra. Moreover, PM5 calculations and B3LYP *ab initio* methods were performed for all compounds. Additionally, analyses of the biological prediction activity spectra for the new esters prepared herein are good examples of *in silico* studies of chemical compounds. We also selected the types of activity that were predicted for a potential compound with the highest probability (focal activities) (Table 1). According to these data the most frequently predicted types of biological activity are: inhibitors glyceryl-ether monooxygenase, acylcarnitine hydrolase, alkylacetylglycerophosphatase, plasmanylethanolamine desaturase, *N*-(acyl)ethanolamine deacylase and protein-disulfide reductase.

**Table 1:** “Probability to be Active” (PA) values for the predicted biological activity of **7**–**9** and **16**–**18**.

Focal Predicted Activity (PA > 0.80)	Compounds					
	7	8	9	16	17	18
Glyceryl-ether mono-oxygenase inhibitor	0.87	0.91	0.93	0.93	0.94	0.95
Acylcarnitine hydrolase inhibitor	-	-	0.81	0.83	0.91	0.94
Alkylacetylglycerophosphatase inhibitor	-	-	-	0.82	0.90	0.86
Plasmanylethanolamine desaturase inhibitor	-	-	-	-	0.71	0.78
CYP3A4 substrate	-	-	-	-	0.70	0.73
*N*-(acyl)ethanolamine-deacylase inhibitor	-	-	-	-	0.73	0.76
Protein-disulfide reductase inhibitor	-	-	0.72	-	0.75	-
Alkenylglycerophosphocholine hydrolase inhibitor	-	-	-	-	0.80	-
Oxidoreductase inhibitor	0.87	0.77	-	-	-	-
Alcohol *O*-acetyl-transferase inhibitor	0.88	-	-	-	-	-
DELTA14-sterol reductase inhibitor	-	0.73	-	-	-	-
Alkylacetylglycerophosphatase inhibitor	-	-	0.84	-	-	-
Antieczematic	-	-	-	0.72	-	-
Glucan endo-1,3-β-D-glucosidase inhibitor	-	-	-	-	0.73	-
D-lactaldehyde dehydrogenase inhibitor	-	-	-	-	-	0.74

**Figure 1:**
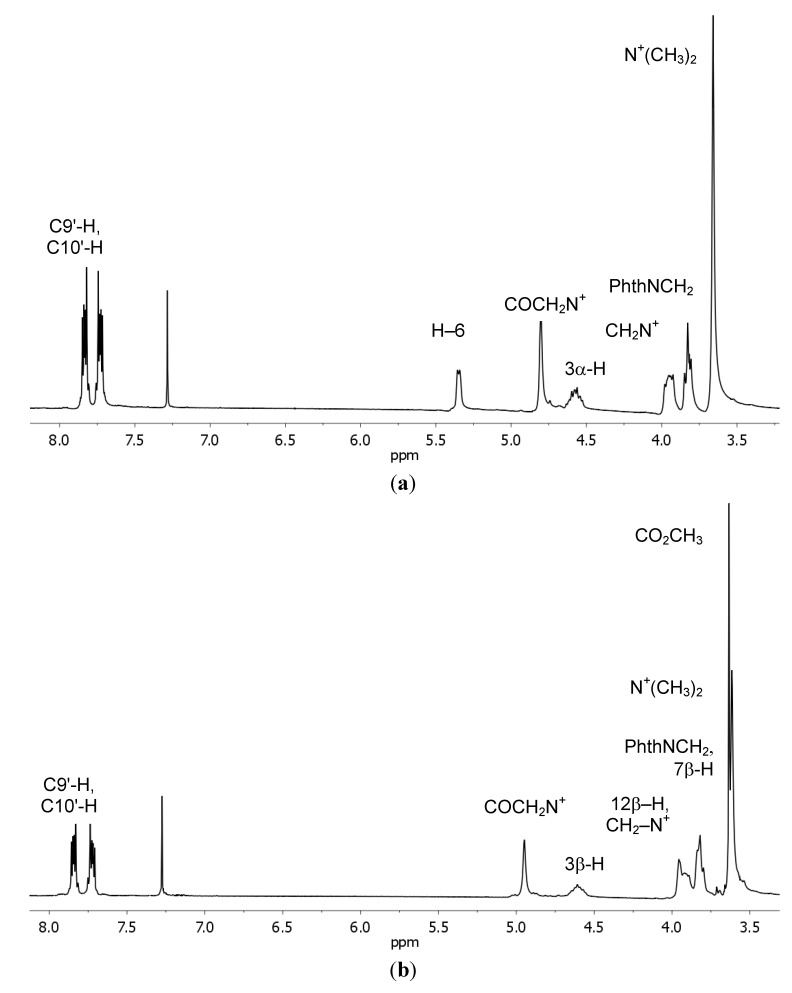
^1^H-NMR spectra in the region (8.0–3.5 ppm) of the most characteristic signals of conjugates **8** (**a**) and **18** (**b**).

The ^1^H-NMR spectra of compounds **7**–**9** show characteristic multiplets in the range 4.96–4.48 ppm assigned to the C3α–H protons of the sterol skeleton. Similarly, compounds **16**–**18** exhibit multiplets in the range 4.92–4.55 ppm which are assigned to the C3β–H protons of the bile acid skeleton (Figure 1). A proton singlet at 0.83 ppm for **7** and singlets in the range of 0.68–0.64 ppm for other conjugates are assigned to CH_3_–18. The other singlets ranging from 1.04–0.79 ppm, 0.91 ppm and 0.89 ppm are assigned to CH_3_–19 for **7**–**9**, **17** and **18**, respectively. In the case of compound **16** signal of the CH_3_–19 group and CH_3_–21 group form the multiplet. The characteristic doublets of CH_3_–21, with the exception of conjugate **16**, are observed at 0.98–0.90 ppm and are assigned to conjugates **7**–**10**, **17** and **18**.

The characteristic doublets of the ergosterol-substituted derivatives are observed at 1.01 ppm for CH_3_–28 group and 0.84 ppm for CH_3_–27 and CH_3_–26 groups, respectively. The ^1^H-NMR spectra of conjugates **8** and **9** show doublets at 0.87 and doublet of doublets at 0.86 ppm of the C(26) and C(27) methyl group. In the ^1^H-NMR spectra of sterol conjugates **7**–**9** and bile acid conjugates **16**–**18** a signal of protons of the COCH_2_N^+^ group in the range 5.35–4.55 ppm is observed. The peaks of six methyl protons of the N^+^(CH_3_)_2_ and two methylene protons of the N^+^CH_2_ appear as singlets and multiplets or broad singlets in the range 3.69–3.61 ppm and 4.96–3.87 ppm, respectively. Two methylene protons of attached to the phthalimide ring–N–CH_2_ group are seen as a broad singlets in the 3.83–3.82 ppm range.

The ^13^C-NMR spectra of conjugates **7**–**9** and **16**–**18** in CDCl_3_ show characteristic signals at 15.80 ppm (**7**), 12.66–11.79 ppm (**8**, **9**, **16**–**18**), and 19.19–17.25 ppm (**7**, **8**, **16**–**18**), 12.18 ppm (**9**) which are assigned to CH_3_–18 and CH_3_–19, respectively. Carbon atoms of the CH_3_–21 group for all conjugates give signals in the ranges 22.55–18.621 ppm. Characteristic shifts of methyl groups present in the sterol side chain (CH_3_–26 and CH_3_–27) are positioned in the range 22.76–19.94 ppm and 22.52–19.61 ppm, respectively. The carbon atoms of the CO_2_CH_3_ unit resonated in the range of 174.77–174.72 ppm and 52.49–51.45 ppm, assigned to CO_2_ and CH_3_, respectively.

Two important signals for C(1')=O and C(3)–O lie at 164.25–163.87 ppm and 77.97–74.87 ppm, respectively. The spectra of all conjugates show two diagnostic signals associated with CH_2_ atoms in N^+^–C(2')H_2_–CO and N^+^–C(4')H_2_ groups. The carbon atoms of the first group are observed at 62.25–60.67 ppm and carbon atoms of the second group lie at 61.50–61.05 ppm for **8**, **9** and **16**–**18** and 57.03 ppm for **7**, respectively. The carbon atoms of N^+^(CH_3_)_2_ group appear in the range of 53.16–51.42 ppm. The carbon atoms of (C(7')=O)_2_N–C(6')H_2_ group resonate in the range of 168.34–168.09 ppm and 62.25–60.67 ppm assigned to C(7')=O and C(6')H_2_, respectively.

The proton chemical shift assignments of *N,N*-dimethyl-(3β-acetate-cholest-5-ene)-3-phthal-imidopropylammonium bromide (**8**) (Table 2) are based on 2D COSY experiments, in which the proton-proton connectivity is observed through the off-diagonal peaks in the counter plot. 

**Table 2:** Chemical shifts (δ, ppm) in D_2_O and calculating GIAO nuclear magnetic shielding tensors (σ_cal_) for *N,N*-dimethyl-(3β-acetate-cholest-5-ene)-3-phthalimido- propylammonium bromide **(8)**. The predicted GIAO chemical shifts were computed from the linear equation δ_exp_ = a + b σ_calc_ with a and b determined from the fit the experimental data.

	δ_exp._	δ_calc_	σ_calc_		δ_exp._	δ_calc_	σ_calc_
C1'	163.90	160.96	98.55	H2'	4.80	5.94	26.538
C2'	62.05	66.51	168.03	H3'	3.66	3.58	28.992
C3'	52.38	44.94	183.90	H4'	3.95	4.94	27.566
C4'	61.05	58.56	173.88	H5'	-	-	30.748
C5'	23.76	20.94	201.56	H6'	3.83	4.16	28.389
C6'	37.63	44.15	184.48	H9'	7.83	7.08	25.346
C7'	168.12	154.39	105.71	H10'	7.77	7.00	25.430
C8'	131.72	134.68	117.88	H3	3.66	3.58	28.987
C9'	123.49	128.37	122.52	H6	5.35	5.04	27.472
C10'	134.22	137.31	115.95	H18	0.68	0.80	32.113
C3	76.57	78.28	159.37	H19	0.99	0.82	31.924
C5	138.76	138.26	115.25	H21	0.92	0.58	32.112
C6	123.33	127.64	123.06	H26,27	0.87	0.76	31.924
C18	11.79	9.93	209.66	-	-	-	-
C19	19.19	35.13	191.12	-	-	-	-
C21	18.65	25.79	197.99	-	-	-	-
C26	22.76	14.29	206.45	-	-	-	-
C27	22.50	11.67	208.38	-	-	-	-
a ^a^	-	-	294.9163	-	-	31.4274	-
b ^b^	-	-	-1.3593	-	-	-0.9606	-
r^2 c^	-	-	0.9831	-	-	0.9443	-

^a^ intercept; *^b^* slope; *^c^* correlation coefficient.

**Figure 2:**
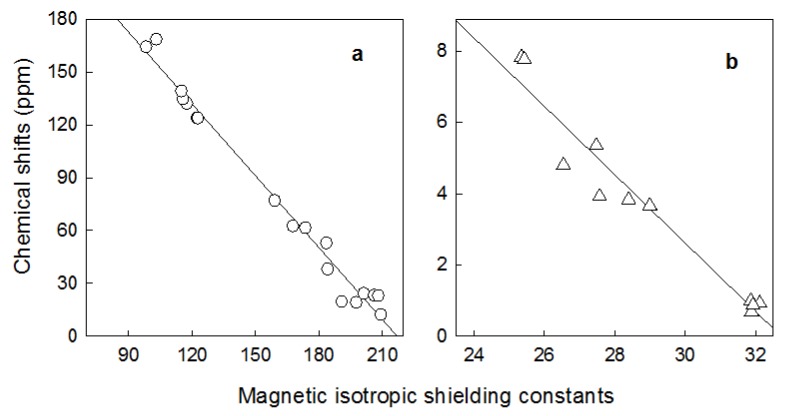
Experimental chemical shifts (δ_exp_.) for *N,N*-dimethyl-(3β-acetate-cholest-5-ene)-3-phthalimidopropylammonium bromide (**8**) *vs.* isotropic magnetic shielding constants (σ_calc_) from the GIAO/B3LYP/6-31G(d,p) calculations; (**a**) carbons-13 and (**b**) protons.

The relations between the experimental ^1^H- and ^13^C-NMR chemical shifts (δ_exp_) and the Gauge-Independent Atomic Orbitals (GIAO) isotropic magnetic shielding tensors (σ_calc_) for **8** are shown in Figure 2. Both correlations are linear, described by the equation: δ_exp._ = a + bσ_calc_. The a and b parameters are given in Table 2. The very good correlation coefficients (r^2^ = 0.9379) for ^1^H and (r^2^ = 0.9984) for ^13^C correlations of *N*,*N*-di-methyl-(3β-acetate-cholest-5-ene)-3-phthalimidopropyl-ammonium bromide confirm the optimized geometry of **8**.

The correlation between the experimental chemical shifts and calculated isotropic screening constants are better for carbon atoms than for protons. The protons are located on the periphery of the molecule and thus they are exposed to stronger interactions with solvent than carbon atoms, which are more hidden inside of structure. The differences between the exact values of the calculated and experimental shifts for protons are probably due to the fact that the shifts are calculated for single molecules in gas phase, whereas experimental values are due to the condensed phase. For this reason the agreement between the experimental and the calculated data for protons are worse than for carbons.

**Figure 3:**
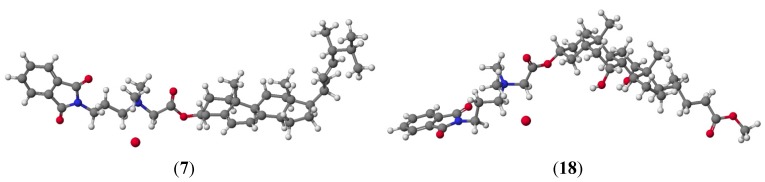
Molecular models of representative compounds **7** and **18** calculated by the PM5 method.

PM5 semiempirical calculations were performed using the WinMopac 2003 program and B3LYP calculation are performed using the GAUSSIAN 03 program package with the 6-31G(d,p) basis set. The final heat of formation (HOF) and energies for the sterols **1**–**3**, bile acids **10**–**12** as well as their conjugates **7**–**9** and **16**–**18** is presented in Table 3. Representative conjugates of sterol **7** and bile acid **18** are shown in Figure 3.

**Table 3:** Heat of formation (HOF) [kcal/mol] and energy [a.u.] of sterols (**1**–**3**), bile acids (**10**–**12**) and their conjugates (**7**–**9**, **16**–**18**).

Compound	Heat of Formation	ΔHOF	Energy	ΔEnergy
	[kcal/mol]	[kcal/mol]	[a.u.]	[a.u.]
1	-97.1208	-	-1113.175477	-
2	-140.1058	-	-1118.205536	-
3	-162.7945	-	-1119.450757	-
7	-177.1668	-80.0460	-4569.562486	-3456.387009
8	-220.6382	-80.5324	-4570.807880	-3452.602344
9	-243.3231	-80.5286	-4605.917678	-3486.466921
10	-236.1585	-	-1204.997432	-
11	-278.4616	-	-1263.040016	-
12	-318.1685	-	-1337.190617	-
16	-306.6215	-70.4630	-4640.248926	-3435.251494
17	-348.9986	-70.5370	−4714.391510	-3451.351494
18	-388.2588	−70.0903	-4788.548913	-3451.358296

ΔHOF = HOF_conjugates (**7**–**9**)_ – HOF_sterols (**1**–**3**)__; _ΔHOF = HOF_conjugates (**16-18**)_ – HOF_bile acids (**10**–**12**)_; ΔEnergy = Energy_conjugates (**7**–**9**)_ – Energy_sterols (**1**–**3**)__; _ΔEnergy = Energy_conjugates (**16**–**18**)_ – Energy_bile acids (**10**–**12**)_.

The lowest values of HOF for sterols are observed for cholestanol **3** and its conjugate **9**, where there are no double bonds which stabilize the molecule and hinder its reactivity, in contrast to conjugates of ergosterol **7** and cholesterol **8** where the double bonds increase the reactivity of the molecule, thereby increasing values of HOF. The HOF relationship for methyl esters of bile acids **10**–**12** and their corresponding conjugates **16**–**18** can be explained in a similar manner. In this case, the number of hydroxyl groups in the steroid skeleton lowers the value of the determinant of HOF. This spatial arrangement of bile acids can facilitate the formation of stable host-guest complexes. These complexes may be stabilized by hydrogen bonding or electrostatic interactions that arise from the number of hydroxyl groups in the bile acid molecule. Similar correlations have been observed using the B3LYP method.

**Figure 4:**
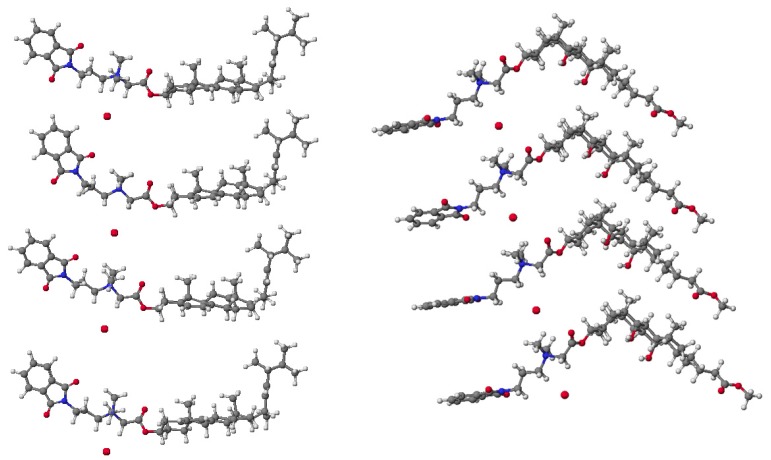
Molecular models of conjugates **7** and **18** calculated by the PM5 method.

The spatial arrangement and interaction of the conjugates **7** and **18** are shown in Figure 4. The final heat of formation is –1249.429 kcal/mol for **7** and –1358.893 kcal/mol for **18** and the distances between the quaternary nitrogen and the anion bromide are 4.34 Å and 4.33 Å, respectively. Compensation charges occurs only through intermolecular electrostatic interaction. This is a very good confirmation of the conclusion that interactions reduce HOF. The dipole moments and selected geometry parameters were calculated at the PM5 and B3LYP /6-31G(d,p) level of theory are presented in Table 4.

**Table 4:** Calculated structural parameters (B3LYP\6-31G(d,p), **PM5**) for sterol conjugates **7**–**9** as well as bile acid conjugates **16**–**18**.

Parameters	7	8	9	16	17	18
Dipole moments (Debye)	9.0817	8.9725	8.8533	8.9578	8.4897	7.7646
Bond lengths [Å]						
N(1)-C(7')	1.451	1.451	1.451	1.459	1.459	1.459
	1.428	1.427	1.428	1.431	1.432	1.430
N(1)-C(3')	1.495	1.494	1.494	1.492	1.492	1.451
	1.471	1.471	1.471	1.469	1.469	1.469
C(1')-O(3)	1.261	1.260	1.260	1.262	1.263	1.262
	1.219	1.220	1.219	1.220	1.220	1.220
N(2)-C(4')	1.573	1.570	1.573	1.572	1.572	1.571
	1.546	1.546	1.546	1.543	1.544	1.543
N(2)-C(2')	1.570	1.574	1.570	1.571	1.572	1.571
	1.527	1.527	1.527	1.525	1.525	1.525
C(7')-O(1)	1.260	1.261	1.260	1.255	1.255	1.255
	1.210	1.210	1.210	1.208	1.208	1.208
Bond angles [°]						
C(6')-C(5')-C(4')	111.5	112.0	112.2	108.8	109.1	108.8
	110.8	110.7	110.9	108.0	108.1	107.7
N(1)-C(6')-C(5')	112.2	112.1	112.1	111.5	111.3	111.5
	110.4	110.5	110.4	110.4	110.5	110.8
C(4')-N(2)-C(2')	105.3	105.4	105.5	105.6	105.8	105.6
	105.6	105.5	111.5	105.6	105.6	105.4
N(2)-C(2')-C(1')	111.6	111.6	111.5	111.3	111.1	111.4
	114.2	114.0	114.0	113.9	113.9	114.0
C(2')-C(1')-O(3)	106.7	106.8	106.9	107.6	102.1	107.6
	109.2	109.4	109.4	109.6	109.1	109.5
Torsion angles [°]						
C(7')-N(1)-C(6')-C(5')	−58.1	−60.0	−60.8	58.3	57.3	58.5
	−82.2	−80.3	−79.4	86.8	85.5	77.6
C(8')-C(7')-N(1)-C(6')	−177.9	−178.5	−178.8	178.2	178.9	178.1
	179.9	−176.9	−178.0	−177.8	−178.1	178.1
C(5')-C(4')-N(2)-C(2')	170.0	171.4	172.1	178.3	178.0	178.2
	172.9	172.1	172.4	−169.2	−168.5	−170.6
C(3')-N(2)-C(2')-C(1')	53.6	56.3	56.1	56.3	56.4	56.5
	61.7	53.1	52.7	55.6	56.2	55.1
C(6')-C(5')-C(4')-N(2)	−99.3	−98.4	−98.0	−179.8	179.1	179.8
	−104.8	−103.8	−103.2	−167.7	−167.3	−172.0
Hydrogen bonds and short contacts lengths						
Distances [Å]						
C(4')-H···Br	3.149	3.147	3.147	3.157	3.159	3.159
	3.283	3.288	3.288	3.434	3.291	3.303
C(2')-H···Br	3.077	3.076	3.075	3.067	3.064	3.068
	3.075	3.080	3.075	3.051	3.045	3.303
N(2)···Br	3.370	3.372	3.373	3.369	3.368	3.370
	3.459	3.464	3.463	3.458	3.463	3.459
Angles [deg]						
C(4')-H···Br	153.0	153.6	153.9	153.5	153.3	153.4
	144.1	143.8	143.9	139.1	138.2	139.4
C(2')-H···Br	157.7	158.0	158.0	158.4	158.5	158.4
	149.6	150.5	150.1	152.0	153.1	152.5

**Figure 5:**
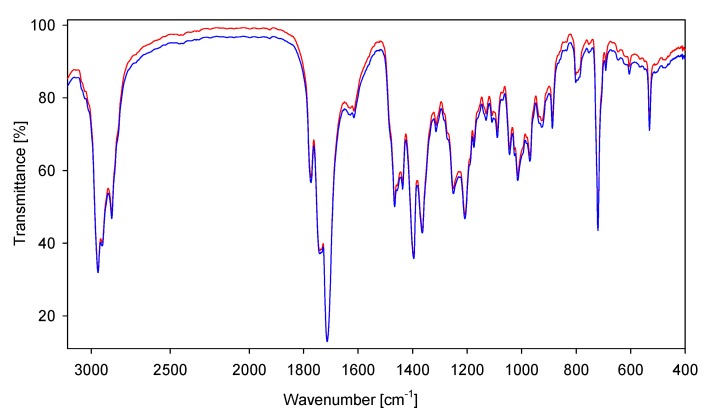
FT-IR spectra of sterol conjugates **7** (red) and **8** (blue) in the 3,100–400 cm^−1^ region.

**Figure 6:**
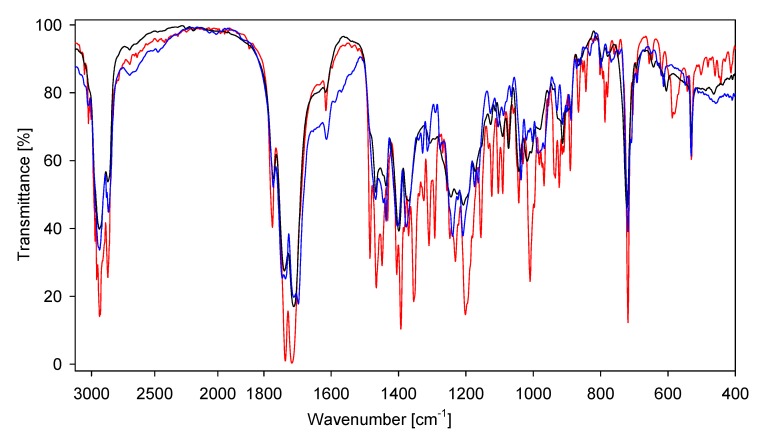
FT-IR spectra of bile acid conjugates **16** (blue), **17** (red), **18** (black) in the 3100–400 cm^−1^ region

The calculated bond lengths and bond angles for **7**–**9** and **16**–**18** optimized by the PM5 and B3LYP methods are quite similar, however the bond lengths N(2)-C(2') and N(2)-C(4') are different. Also the torsion angles calculated by the PM5 and B3LYP methods are slightly different, especially the C(5')-C(4')-N(2)-C(2') angle for fatty acid conjugates **16**–**18**. This shows a crucial role of electrostatic interaction between oppositely charged groups in the structure of the investigated compounds (Table 4). Hydrogen bonds and short contact lengths distances are shorter for compounds calculated by B3LYP method. These data prove that in the gas phase the type of quantum chemical methods used play an important role in the molecular structure of ionic compounds. The solid-state IR spectra of sterols and bile acids conjugates are shown in Figure 5 and Figure 6, respectively.

**Figure 7:**
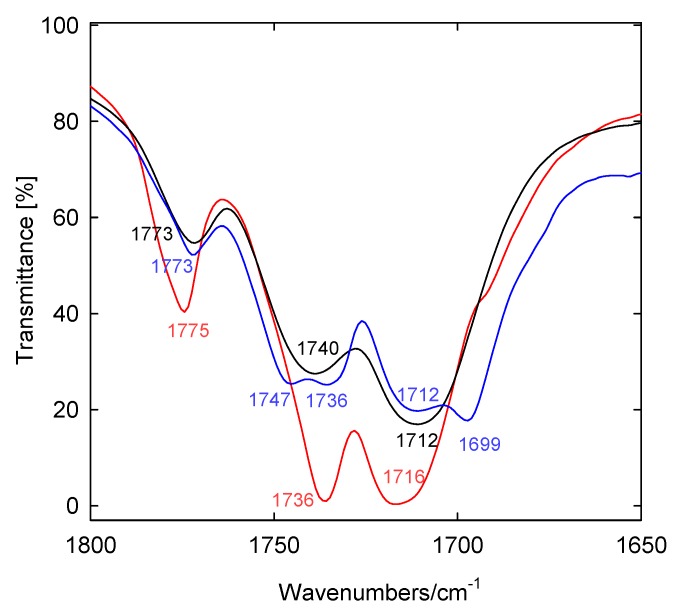
FT-IR spectra of bile acid conjugates in the carbonyl group region **16** (blue), **17** (red), **18** (black).

**Figure 8:**
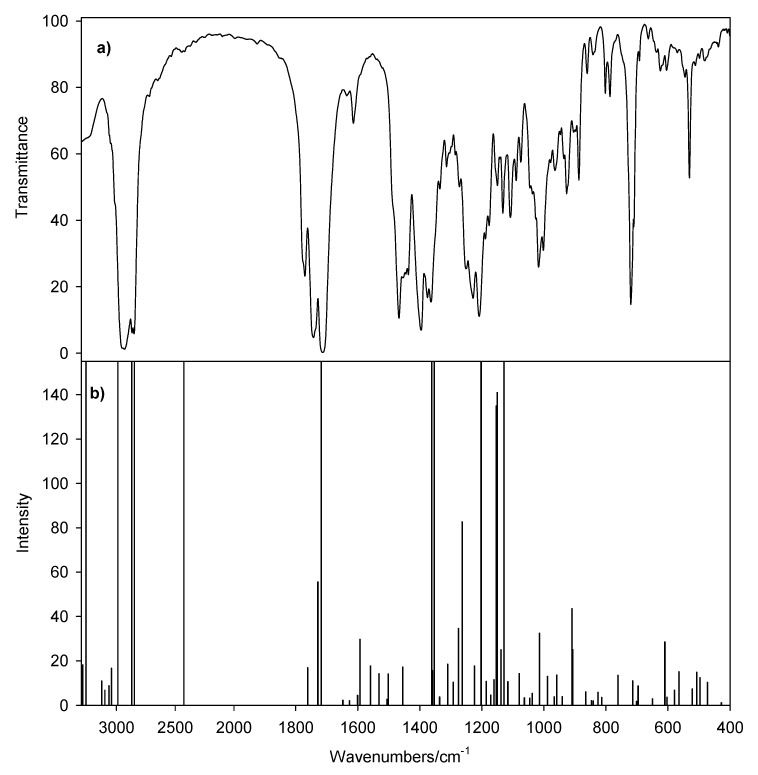
Spectrum of *N,N*-dimethyl-(3β-acetate-5β-cholestan)-3-phthalimidopropyl-ammonium bromide (**9**); (**a**) FT-IR (**b**) calculated scaled FT-IR spectrum.

The FT-IR spectra of conjugates show characteristic bands at 1,775–1,772 cm^−1^ which are due to the asymmetric carbonyl group ν_as_C=O stretching vibrations in a phthalimide moiety (Figure 5, Figure 6, Figure 7 and Figure 8) [48,49]. The symmetric ν_s_C=O stretching vibration appears in the FT-IR spectrum as an intense and broad nonsymmetrical band at 1,716–1,699 cm^−1^ suggesting the small nonequivalence of carbonyl groups in the phthalimide moiety. Moreover strong characteristic bands in the region 1,251–1,240 cm^−1^ are present, which are assigned to the *ν*(C–O). 

The FT-IR spectra of bile acid conjugates (Figure 7, blue line) shows characteristic vibration bands of ν_as_C=O and ν_s_C=O in a phthalimide moiety at 1,773 cm^−1^ and at 1,712 and 1,699 cm^−1^, respectively. In this case the ν_as_C=O and ν_s_C=O bands are more nonsymmetrical in comparison to the carbonyl bands of **17** and **18**. The nonequivalence of ν_as_C=O in the FT-IR spectrum is observed for *N,N*-bis-(phthalimidopropyl)-*N*-propylamine [50]. In contrast to the above examples, in *N,N*-dimethyl-3-phthalimidopropylammonium hydrochloride monohydrate and *N*-*n*-butyltetrachlorophthalimide no split of the carbonyl bands in FT-IR spectra were observed, in spite of the different interactions of each carbonyl group in the supramolecular structure [51,52]. The ν(COO) stretching vibrations of carboxy groups are observed at 1,747–1,736 cm^−1^ (Figure 7).

The room-temperature solid-state FT-IR and the calculated spectrum of **9** are shown in Figure 8. The band frequencies, relative intensities and their assignments in the 4,000–400 cm^−1^ range are listed in Table 5. For convenience of comparison, the band intensities for the calculated spectrum are scaled.

**Table 5:** Observed and calculated B3LYP/6-31G(d,p) vibrational frequencies and infrared intensities for *N,N*-dimethyl-(3β-acetate-5β-cholestan)-3-phthalimidopropylammonium bromide (**9**).

IR	IR_calc._	IR_calc_._scaled_	INT	Proposed Assignment
3,462w	3,477	3,344	3.34	nCH
3,459w	3,467	3,333	17.8	nCH
3,380w	3,464	3,330	5.26	nCH
3,304w	3,419	3,286	18.2	nCH_2_
3,243w	3,391	3,259	381	nCH_2_
3,060w	3,252	3,125	10.9	nCH_2_
3,048s	3,225	3,098	6.74	nCH_2_
2,952s	3,189	3,063	8.81	nCH_3_
2,933s	3,167	3,042	16.7	nCH_3_
2,930s	3,111	2,988	485	nCH_2_
2,867s	2,988	2,869	515	nCH_2_
2,851s	2,966	2,847	994	nCH_2_
2,719w	-	-	-	nCH∙∙∙Br
2,649w	2,531	2,486	1982	nCH∙∙∙Br
2,534w	-	-	-	nCH∙∙∙Br
1,770m	1,845	1,761	16.9	n_as_CO
1,742s	1,811	1,728	55.6	nCOO
1,712s	1,800	1,718	190	n_s_CO
1,635vw	1,728	1,650	2.38	nCC
1,614w	1,706	1,627	2.13	nCC
-	1,679	1,600	4.59	nCC
-	1,671	1,593	29.7	nCC
-	1,636	1,558	17.7	nCC
1,467s	1,608	1,532	14.2	nCC
1,454m	1,581	1,505	2.76	nCC, βCH_2_
1,445 m	1,577	1,502	14.1	β_as_CH_3_
1,437m	1,529	1,455	17.2	βCH_2_
1,396s	1,432	1,361	175	βOH
1,375 m	1,430	1,359	15.7	β_s_CH_3_
1,364 m	1,424	1,353	178	nCO
1,335 w	1,406	1,336	3.82	nCC
1,313 w	1,379	1,310	18.5	nCN
1,285 w	1,361	1,292	10.4	nCC
1,272 w	1,343	1,275	34.6	nCC, βCH_2_
1,251 m	1,331	1,263	82.7	nCO
1228 m	1,290	1,224	17.7	βCH_2_
1,209 s	1,268	1,202	232	nCC
1,188 m	1,251	1,186	10.8	nCC
1,176 m	1,236	1,172	4.70	nCC
1,150 w	1,225	1,161	11.5	nCN
1,142 w	1,217	1,153	135	nCN
1,132 w	1,214	1,150	141	βCH
1,108 w	1,202	1,138	25.0	nCN
1,089 w	1,192	1,128	169	nCN
1,074 w	1,179	-	10.6	γCH_2_
1,044 w	1,141	1,116	14.3	γCH_2_
1,037 w	1,124	1,079	3.42	γCH_2_
1,026 w	1,106	1,063	3.28	δCH_2_
1,017 m	1,098	1,045	5.41	βCH_2_
1,001 m	1,074	1,037	32.5	γCH_2_
978 w	1,047	1,014	13.0	βCCC
964 w	1,025	988	3.87	βCO
950 w	1,016	967	13.6	γCH_2_
935 w	998	958	3.96	βCH_2_
927 w	966	941	43.5	γCH_2_
904 w	963	909	25.0	γCH_2_
897 w	920	907	6.08	βCCC
887 w	901	907	2.12	βCCC
860 vw	895	865	2.04	tring
842 vw	879	847	5.89	nCC
802 vw	867	825	3.62	βCCC
787 vw	813	814	13.5	βCCC
720 m	764	761	11.0	βCCC
710 vw	751	714	1.86	βCNC
692 vw	746	701	8.70	βring
663 vw	698	694	3.00	γCH
636 vw	659	612	0.13	γCH
625 vw	657	610	28.5	γCH
605 vw	650	603	3.71	βring
600 vw	625	579	6.76	βNCC
545 vw	610	565	15.1	γCC
531 w	566	522	7.31	βCCC
512 vw	551	508	14.9	tring
498 vw	540	497	12.4	tring	
482 vw	515	472	10.3	γCCC	
438 vw	469	428	1.23	Lattice mode	
419 vw	434	394	11.4	Lattice mode	
409 vw	409	370	9.39	Lattice mode	
-	386	348	5.04	Lattice mode	
-	345	308	22.0	Lattice mode	
-	329	292	57.4	Lattice mode	
-	282	247	3.62	Lattice mode	
-	257	223	10.3	Lattice mode	
-	205	172	5.27	Lattice mode	
-	177	145	1.08	Lattice mode	
-	102	72	1.63	Lattice mode	
-	56	28	2.59	Lattice mode	

The abbreviations are: s: strong; m: medium; w: weak; vw; very weak; ν: stretching; β: in plane bending; δ: deformation; w: wagging; γ: out of plane bending and τ: twisting.

**Figure 9:**
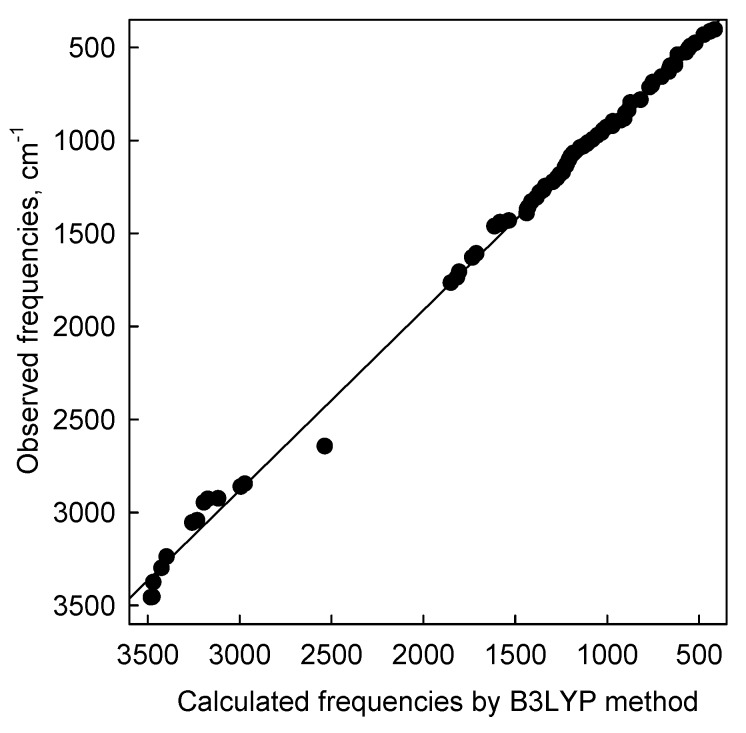
Correlation between the experimental and calculated wavenumbers (cm^−1^) for (**9**); ν_scaled_ = -26.3525 + 0.9689ν_calc._, r^2^ = 0.9973.

The DFT harmonic vibrational wavenumbers are usually higher than the experimental values. However, in this case the overall agreement between the experimental and calculated frequencies for (**9**) is very good (Figure 9).

Any discrepancy noted between the observed and calculated frequency may be due to the fact that the calculations have been done for a single molecule in the gaseous state contrary to the experimental spectrum recorded in the presence of intermolecular interactions. The scaling procedure, as recommended by Palafox was used [53,54]. The scaled IR spectrum is shown in Figure 8b and the predicted frequencies are listed in Table 3 as ν_scaleq_. Scaling of the harmonic vibrational frequencies reproduce the experimental solid-state FT-IR frequencies with the r.m.s. error of 38.1 cm^−1^. The vibrational band assignments of 9 were made using Gauss-View molecular visualization program [55].

## 3. Experimental

### 3.1. General

The NMR spectra were measured with a Varian Mercury 300 MHz NMR spectrometer (Oxford, UK), operating at 300.07 and 75.4614 for ^1^H and ^13^C, respectively. Typical conditions for the proton spectra were: pulse width 32°, acquisition time 5 s, FT size 32 K and digital resolution 0.3 Hz per point, and for the carbon spectra pulse width 60°, FT size 60 K and digital resolution 0.6 Hz per point, the number of scans varied from 1200 to 10,000 per spectrum. The ^13^C and ^1^H chemical shifts were measured in CDCl_3_ relative to an internal standard of TMS. Infrared spectra were recorded in the KBr pellets using a FT-IR Bruker IFS 66 spectrometer (Karlsruhe, Germany). The ESI (electron spray ionization) mass spectra were recorded on a Waters/Micromass (Manchester, UK) ZQ mass spectrometer equipped with a Harvard Apparatus (Saint Laurent, QC, Canada), syringe pump. The sample solutions were prepared in methanol at the concentration of approximately 10^−5^ M. The standard ESI-MS mass spectra were recorded at the cone voltage 110 V. The MALDI (matrix-assisted laser desorption/ionization) mass spectra were recorded on a Waters Maldi Q-Tof Premiere. The sample solutions were prepared in methanol at the concentration of approximately 10^−5^ M. The matrix was 2,5-dihydroxybenzoic acid (gentisic acid) and the standard was β-cyclodextrin (*m/z* 1157.3218). PM5 semiempirical calculations were performed using the WinMopac 2003 program [56,57,58]. The calculations were performed using the GAUSSIAN 03 program package [59] at the B3LYP [60,61,62] levels of theory with the 6-31G(d,p) basis set [63]. The NMR isotropic shielding constants were calculated using the standard Gauge-Independent Atomic Orbital (GIAO) approach of Gaussian 03 [64,65].

### 3.2. Synthesis

The appropriate 3β-bromoacetate of sterols or 3α-bromoacetate of bile acids (0.20 mmol) were dissolved in CH_3_CN (6 mL) under reflux. Then *N*,*N*-dimethyl-3-phthalimidopropylamine (0.24 mmol) was added and the mixture heated under reflux for 3 h. The precipitate was filtered and crystallized from CH_3_CN-EtOH (90:1), to give white solids.

*N,N-Dimethyl-(3β-acetate-ergosta-5,7,22-triene)-3-phthalimidopropylammonium bromide* (**7**): white solid (76%), m.p. 185–187 °C. ^1^H-NMR: δ_H_ 7.85 (bs, 2H, Ar–H), 7.75 (bs, 2H, Ar–H), 5.35 (s, 2H, COCH_2_N^+^), 5.31–5.12 (m, 4H, 6, 7, 22, 23–H), 4.96–4.58 (m, 3H, 3α–H, N^+^CH_2_), 3.82 (bs, 2H, CH_2_–N–phthalimide ring), 3.69 (s, 6H, N^+^(CH_3_)_2_), 1.04 (s, 3H, CH_3_–19), 1.01 (d, *J* = 6,0 Hz, 3H, CH_3_–28), 0.93 (d, *J* = 6.7 Hz, 3H, CH_3_–21), 0.84 (d, *J* = 6.7 Hz, 6H, CH_3_–26 and CH_3_–27), 0.83 (s, 3H, CH_3_–18). ^13^C-NMR: δ_C_ 168.17 (C-7'), 163.98 (C-1'), 140.11 (C-8), 139.96 (C-5), 135.43 (C-22), 134.32, 132.08 (C-23), 131.81, 123.57, 118.11 (C-6), 118.03 (C-7), 74.87 (C-3), 62.40, 61.67, 60.67 (C-2'), 57.03 (C-4'), 53.16 (C-3'), 44.76, 42.79, 40.80, 40.72, 38.86, 36.82, 36.65, 36.44, 36.40, 34.93, 34.03, 33.81, 33.05, 27.58, 27.45, 26.43, 25.10, 25.04, 22.81, 21.77 (C-21), 20.96 (C-19), 19.94, 19.61, 18.28, 18.20, 17.63, 15.80 (C-18). FT-IR (KBr) ν_max_: 2957, 2871, 1773, 1740, 1713, 1466, 1437, 1396, 1365,1250, 1208, 1044, 926. ESI-MS (*m/z*): 670 (100%) [C_43_H_61_N_2_O_4_]^+^. MALDI-MS (*m/z*): 669.9. HRMS: calcd 669.5678 for C_43_H_61_N_2_O_4_; found 669.5630.

*N,N-Dimethyl-(3β-acetate-cholest-5-ene)-3-phthalimidopropylammonium bromide* (**8**): white solid (82%), m.p. 199–200 °C. ^1^H-NMR: δ_H_ 7.85–7.80 (m, 2H, Ar–H), 7.76–7.70 (m, 2H, Ar–H), 5.35 (d, *J* = 4.9 Hz, 1H, 6–H), 4.80 (bs, 2H, COCH_2_N^+^), 4.69–4.48 (m, 1H, 3α–H), 4.00–3.89 (m, 2H, N^+^CH_2_), 3.83 (bs, 2H, CH_2_–N–phthalimide ring), 3.66 (s, 6H, N^+^(CH_3_)_2_), 0.99 (s, 3H, CH_3_–19), 0.92 (d, *J* = 6.4 Hz, 3H, CH_3_–21), 0.87 (dd, *J* = 6.6, 1.4 Hz, 6H, CH_3_–26 and CH_3_–27), 0.68 (s, 1H, CH_3_–18). ^13^C-NMR: δ_C _168.12 (C-7'), 163.90 (C-1'), 138.76 (C-5), 134.22, 131.72, 123.49, 123.33 (C-6), 76.57 (C-3), 62.05 (C-2'), 61.05 (C-4'), 56.60, 56.06, 52.38 (C-3'), 49.93, 42.24, 39.62, 39.44, 37.63, 36.75, 36.44, 36.11, 35.71, 34.63, 31.84, 31.72, 29.64, 28.15, 27.95, 27.42, 24.21, 23.76, 22.76, 22.50, 20.95, 19.19 (C-19), 18.65 (C-21), 11.79 (C-18). FT-IR (KBr) ν_max_: 2943, 2868, 1774, 1737, 1713, 1467, 1637, 1395, 1364, 1252, 1210, 1089, 925. ESI-MS (*m/z*): 660 (100%) [C_42_H_63_N_2_O_4_]^+^. MALDI-MS (*m/z*): 659.9. HRMS: calcd 659.5812 for C_42_H_63_N_2_O_4_; found 659.5802. 

*N,N-Dimethyl-(3β-acetate-5β-cholestan)-3-phthalimidopropylammonium bromide* (**9**): white solid (83%), m.p. 191–192 °C. ^1^H-NMR: δ_H_ 7.83 (d, *J* = 3.90 Hz, 2H, Ar–H), 7.74 (m, 2H, Ar–H), 4.88–4.55 (m, 3H, COCH_2_N^+^, 3α–H), 3.92 (bs, 2H, N^+^CH_2_), 3.83 (bs, 2H, CH_2_–N–phthalimide ring), 3.61 (s, 6H, N^+^(CH_3_)_2_), 0.90 (d, *J* = 6.5 Hz, 3H, CH_3_-21), 0.86 (dd, *J* = 6.7, 1.9 Hz, 6H, CH_3_–26 and CH_3_–27), 0.79 (s, 3H, CH_3_–19), 0.65 (s, 1H, CH_3_–18). ^13^C-NMR: δ_C_ 168.18 (C-7'), 163.89 (C-1'), 134.26, 131.74, 123.51, 76.68 (C-3), 62.25 (C-2'), 61.27 (C-4'), 56.34, 56.21, 54.10, 52.51 (C-3'), 44.62, 42.52, 39.90, 39.45, 36.56, 36.11, 35.74, 35.34, 34.65, 33.55, 31.90, 29.65, 28.44, 28.18, 27.96, 27.13, 24.14, 23.79, 22.78, 22.56, 22.52, 21.15, 18.62 (C-21), 12.18 (C-19), 12.02 (C-18). FT-IR (KBr) ν_max_: 2952, 2867, 1770, 1742, 1712, 1467, 1445, 1396, 1375, 1251, 1089, 927. ESI-MS (*m/z*): 661 (100%) [C_42_H_65_N_2_O_4_]^+^. MALDI-MS (*m/z*): 661.5. HRMS: calcd 661.5905 for C_42_H_65_N_2_O_4_; found 661.5917. 

*N,N-Dimethyl-(methyl litocholate)-3-phthalimidopropylammonium bromide* (**16**): white solid (96%), m.p. 104–105 °C. ^1^H-NMR: δ_H_ 7.86–7.82 (m, 2H, Ar–H), 7.77–7.73 (m, 2H, Ar–H), 4.84–4.67 (m, 3H, 3β–H, COCH_2_N^+^), 3.92 (bs, 2H, N^+^CH_2_), 3.83 (bs, 2H, CH_2_–N–phthalimide ring), 3.66 (s, 9H, CO_2_CH_3_ and N^+^(CH_3_)_2_), 0.91 (m, 6H, CH_3_–19 and CH_3_–21), 0.64 (s, 3H, CH_3_–18). ^13^C-NMR: δ_C_ 174.72 (C-24), 168.09 (C-7'), 163.87 (C-1'), 134.24, 131.73, 123.45, 77.97 (C-3), 62.11 (C-2'), 61.14 (C-4'), 56.15, 55.79, 52.49 (C-3'), 51.42 (C-25), 43.04, 42.64, 41.89, 40.23, 39.89, 35.68, 35.28, 35.02, 34.81, 34.61, 34.45, 31.76, 31.00, 30.93, 28.10, 26.84, 26.23, 26.13, 24.09, 23.16, 22.53, 20.76 (C-21), 18.21 (C-19), 11.97 (C-18). FT-IR (KBr) ν_max_: 2937, 2867, 1773, 1746, 1737, 1712, 1698, 1466, 1438, 1404, 1378, 1210, 1088, 930. ESI-MS (*m/z*): 664 (100%) [C_40_H_59_N_2_O_6_]^+^. MALDI-MS (*m/z*): 663.5. HRMS: calcd 663.5361 for C_40_H_59_N_2_O_6_; found 663.5396. 

*N,N-Dimethyl-(methyl deoxycholate)-3-phthalimidopropylammonium bromide* (**17**): white solid (90%), m.p. 170–172 °C. ^1^H-NMR: δ_H_ 7.87–7.83 (m, 2H, Ar–H), 7.76–7.72 (m, 2H, Ar–H), 4.92–4.67 (m, 3H, 3β–H, COCH_2_N^+^), 3.98 (s, 1H, 12β–H), 3.96–3.87 (m, 2H, CH_2_–N^+^), 3.82 (bs, 2H, CH_2_–N–phthalimide ring), 3.63 (s, 9H, CO_2_CH_3_ and N^+^(CH_3_)_2_), 0.98 (d, *J* = 5.7 Hz, 3H, CH_3_–21), 0.91 (s, 3H, CH_3_–19), 0.66 (s, 3H, CH_3_–18). ^13^C-NMR: δ_C_ 174.75 (C-24), 168.30 (C-7'), 164.00 (C-1'), 134.26, 131.76, 123.55, 77.66 (C-3), 72.81 (C-12), 62.07 (C-2'), 61.39 (C-4'), 52.55 (C-3'), 51.45 (C-25), 48.04, 47.08, 46.40, 41.81, 35.86, 35.13, 34.71, 34.03, 33.37, 31.62, 31.07, 30.90, 28.61, 27.41, 26.77, 26.09, 25.97, 23.62, 22.87, 22.55 (C-21), 17.26 (C-19), 12.66 (C-18). FT-IR (KBr) ν_max_: 2955, 2870, 1774, 1736, 1716, 1485, 1466, 1405, 1393, 1201, 1091, 967. ESI-MS (*m/z*): 680 (100%) [C_40_H_59_N_2_O_7_]^+^. MALDI-MS (*m/z*): 679.5. HRMS: calcd 679.5358 for C_40_H_59_N_2_O_7_; found 679.5341.

*N,N-Dimethyl-(methyl cholate)-3-phthalimidopropylammonium bromide* (**18**): white solid (85%), m.p. 204–205 °C. ^1^H-NMR: δ_H_ 7.86–7.83 (m, 2H, Ar–H), 7.76–7.69 (m, 2H, Ar–H), 4.95 (s, 2H, COCH_2_N^+^), 4.66–4.55 (m, 1H, 3β–H), 3.97 (bs, 1H, 12β–H), 3.92–3.89 (m, 2H, CH_2_–N^+^), 3.82 (bs, 2H, CH_2_–N–phthalimide ring), 3.80 (bs, 1H, 7β–H), 3.63 (s, 3H, CO_2_CH_3_,), 3.62 (s, 6H, N^+^(CH_3_)_2_), 0.97 (d, *J* = 5.8 Hz, 3H, CH_3_–21), 0.89 (s, 3H, CH_3_–19), 0.66 (s, 3H, CH_3_–18). ^13^C-NMR: δ_C_ 174.77 (C-24), 168.34 (C-7'), 164.25 (C-1'), 134.19, 131.78, 123.49, 76.57 (C-3), 72.62 (C-12), 67.75 (C-7), 61.86 (C-2'), 61.50 (C-4'), 52.50 (C-3'), 51.41 (C-25), 46.95, 46.35, 41.89, 41.22, 39.54, 35.26, 34.86, 34.66, 34.37, 31.13, 30.91, 28.37, 27.45, 26.68, 26.30, 23.13, 22.55, 22.32 (C-21), 17.25 (C-19), 12.47 (C-18). FT-IR (KBr) ν_max_: 2938, 2870, 1772, 1739, 1711, 1468, 1437, 1399, 1370, 1208, 1073, 953. ESI-MS (*m/z*): 696 (100%) [C_40_H_59_N_2_O_8_]^+^. MALDI-MS (*m/z*): 695.5. HRMS: calcd 695.5192 for C_40_H_59_N_2_O_8_; found 695.5242.

## 4. Conclusions

In conclusion, six new quaternary *N*,*N*-dimethyl-3-phthalimidopropylammonium conjugates of sterols (compounds **7**–**9**) and bile acids (compounds **16**–**18**) were prepared by the reactions of ergosteryl 3β-bromoacetate, cholesteryl 3β-bromoacetate, dihydrocholesteryl 3β-bromoacetate as well as methyl litocholate 3α-bromoacetate, methyl deoxycholate 3α-bromoacetate and methyl cholate 3α-bromoacetate, with *N*,*N*-dimethyl-3-phthalimidopropylamine in acetonitrile. These new compounds were characterized by spectroscopic and molecular structure methods. The obtained conjugates may find applications in molecular recognition and in pharmacology, especially as compounds with a high antimicrobial activity.
